# Conjugate Addition of Nucleophiles to the Vinyl Function of 2-Chloro-4-vinylpyrimidine Derivatives

**DOI:** 10.3390/molecules15031973

**Published:** 2010-03-19

**Authors:** Elizabeth Raux, Jeffrey Klenc, Ava Blake, Jarosław Sączewski, Lucjan Strekowski

**Affiliations:** Department of Chemistry, Georgia State University, Atlanta, Georgia 30302-4098, USA

**Keywords:** conjugate addition, vinylpyrimidine, vinylquinazoline

## Abstract

Conjugate addition reaction of various nucleophiles across the vinyl group of 2-chloro-4-vinylpyrimidine, 2-chloro-4-(1-phenylvinyl)pyrimidine and 2-chloro-4-vinyl-quinazoline provides the corresponding 2-chloro-4-(2-substituted ethyl)pyrimidines and 2-chloro-4-(2-substituted ethyl)quinazolines. Treatment of these products, without isolation, with *N*-methylpiperazine results in nucleophilic displacement of chloride and yields the corresponding 2,4-disubstituted pyrimidines and quinazolines.

## 1. Introduction 

Few addition reactions across the vinyl group of vinyl-substituted heterocyclic compounds are known. Photoirradiation of 2-vinylpyridine in acidic alcohol affords alkyl 2-(2-pyridyl)ethyl ether [1]. The treatment of 2-vinylpyridine or 4-vinylpyridine under acidic conditions with various amines yield the corresponding aminoethyl-substituted pyridines [2]. Ethyl methyl sulfones are produced upon treatment of vinyl-substituted pyridines, pyrimidines and other diazines with sodium methanesulfinate under acidic conditions [3]. The reaction of indole with the vinyl-substituted substrates yields 2-(indol-3-yl)ethyl derivatives when conducted under acidic conditions [4]. On the other hand, 2-(indol-1-yl)ethyl substituted heteroaromatic compounds are produced for the reactions conducted under basic conditions at elevated temperatures [4]. Finally, various alkylthiols and arylthiols undergo a facile addition reaction with 4-vinylquinazoline to give the corresponding 4-[2-(alkyl/arylthio)-ethyl]quinazolines [5]. 

This report pertains to the conjugate addition reaction of N-, O-, S-, and C-centered nucleophiles across the vinyl function of 2-chloro-4-vinylpyrimidine (**2**), 2-chloro-4-(1-phenylvinyl)pyrimidine (**3**), and 2-chloro-4-vinylquinazoline (**19**). These vinyl-bearing substrates are readily available by the addition reaction of a vinyllithium reagent to the formal C4=N3 bond of 2-chloropyrimidine and 2-chloroquinazoline, followed by aromatization of the resultant adduct [4,5,6,7]. Another approach to similar compounds involves dehydration of an alcohol to the vinyl group [7]. The study described in this paper provides a facile modification of the pyrimidine and quinazoline systems with diverse substituents. More specifically, the selective nucleophile addition reaction across the vinyl group can be followed by nucleophilic displacement reaction of the 2-chloride substituent in the intermediate product to give a disubstituted pyrimidine derivative. Similar compounds have been shown to interact selectively with various serotonin receptors of the central nervous system (CNS) and have shown promise for the development as practical CNS drugs [6,8,9,10]. 

## 2. Results and Discussion 

**Scheme 1:**
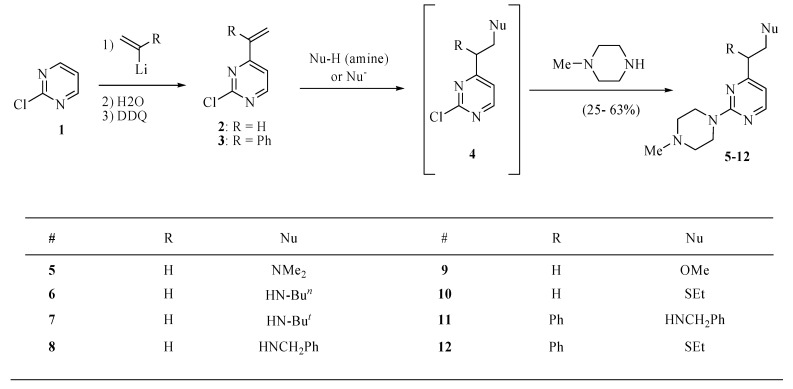
Synthesis of pyrimidines **5–12**.

Improved procedures for the preparation of 2-chloro-4-vinylpyrimidine (**2**, Scheme 1) [11] and 2-chloro-4-vinylquinazoline (**19**, Scheme 4) [5] have been reported by us recently [6]. 2-Chloro-4-(1-phenylvinyl)pyrimidine (**3**, Scheme 1) is a new compound. For the synthesis of **3**, α-bromostyrene was allowed to react with *n*-butyllithium and this generated lithium reagent was treated with 2- chloropyrimidine (**1**). The resultant adduct was then aromatized by the reaction with 2,3-dichloro-5,6-dicyanoquinone (DDQ) to give the desired product **3**. 

As can be seen from Scheme 1, a variety of *N-, O-,* and *S*-centered nucleophiles undergo selective addition reactions across the vinyl function of **2** and **3**. A subsequent treatment of the crude reaction mixture containing the corresponding adducts **4** with *N*-methylpiperazine furnished the corresponding products **5**–**12**. The yields ranged from a low of 25% for the ethanethiol derivative **10** to a high of 63% for the benzylamino derivative **8**. It should be noted that these yields are for the analytically pure products obtained in the indicated two-step transformations starting with 2-chloropyrimidine (**1**). In all cases, compounds **5–12** were the major products that were easily isolated by chromatography. The reaction also produced many minor products that could not be isolated for identification and a substantial amount of tar. The high selectivity of the first addition reaction across the vinyl function was consistent with a GC-MS analysis of all crude mixtures containing **4**, which indicated the presence of the chlorine atom in the major product. In one case the intermediate product **4** (R = Ph, Nu = MeO) was isolated and characterized. 

**Scheme 2:**
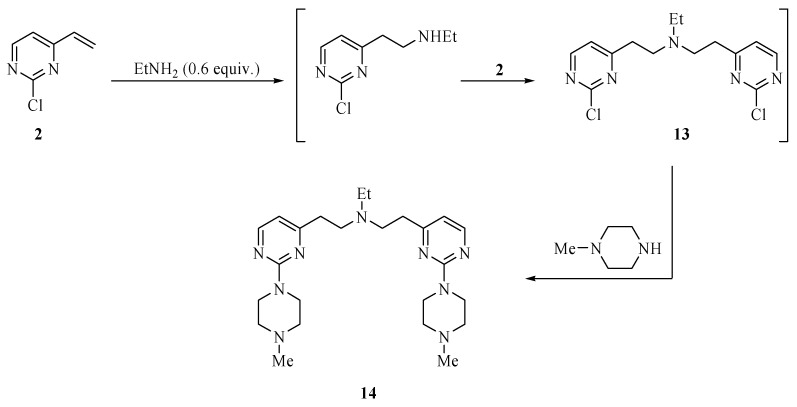
Synthesis of pyrimidine **14**.

The chemistry of Scheme 1 was conducted using equimolar amounts of the vinylpyrimidine and nucleophile substrates. It was shown that the rate of the vinyl addition reaction is greater than that of the chloride displacement. Additional evidence for the selective nucleophile addition is presented in Scheme 2 for the treatment of **2** with 0.55 equiv. of ethylamine followed by standard treatment of the presumed intermediate product **13** with *N*-methylpiperazine. The final product **14** was the only major component of the crude mixture and was isolated in an analytically pure form in a 38% yield. 

The treatment of **2** or **3** with lithium reagents such as methyllithium and phenyllithium produced a large number of products, none of them major. These reactions were not simplified when conducted in the presence of cuprous iodide. However, analogous reactions with the corresponding Grignard reagents gave less complicated mixtures, which upon treatment with *N*-methylpiperazine yielded the corresponding products **15–17** that were isolated and characterized. Interestingly, the low yields of **15**–**17** (<10%) were increased to the range 40–77% when the initial addition reactions were conducted in the presence of cuprous iodide. Under the optimized conditions the ratio of Grignard reagent to CuI is 2:1. The observed effect of CuI is consistent with a mechanism the initial step of which involves single electron transfer (SET) from a cuprate intermediate product to the vinylpyrimidine substrate [11,12]. It is not certain, however, whether a SET process is operative in the addition reaction of heteroatom-centered nucleophiles. The structures of **15–17** are fully consistent with their high-resolution mass, ^1^H-NMR, and ^13^C -NMR spectra. In particular, the absorption pattern for a propyl group, observed in the ^1^H-NMR spectra of **15** and **17**, shows the selective addition of the methyl Grignard reagent to the vinyl function of **2** and **3**, respectively.

**Scheme 4:**
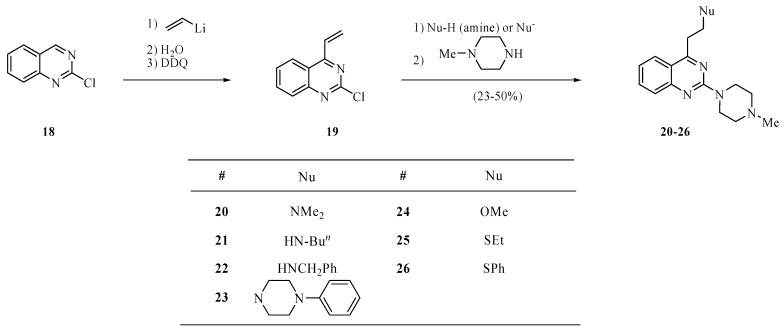
Synthesis of quinazolines **20–26**.

The synthesis of 2-chloro-4-vinylquinazoline (**19**) based on the addition reaction of vinyllithium with 2-chloroquinazoline (**18**, Scheme 4) has been reported by us recently [6]. We now report that, as with vinylpyrimidines, the addition reaction of nucleophiles with the vinyl function of **19** is highly selective. The presence of a chlorine atom in the intermediate adducts could be observed by mass spectrometry. As with the pyrimidines, treatment of the intermediate products with *N*-methylpiperazine furnishes the corresponding disubstituted quinazolines.

## 3. Experimental 

### 3.1. General

All organometallic reactions were conducted under a nitrogen atmosphere in tetrahydrofuran distilled from sodium benzophenone ketyl immediately before use. Final products were purified on a chromatotron using silica gel-coated rotors (2.0 mm). Hydrobromide and hydrochloride salts of the piperazine products were obtained by using a general procedure [9,10] and the salts were crystallized from 95% ethanol. In several cases it was necessary to dilute the ethanolic solution with ether to induce crystallization. Melting points are not corrected. ^1^H-NMR and ^13^C-NMR spectra were recorded at 400 MHz and 100 MHz, respectively, on a Bruker Avance insturment. 

### 3.2. Organometallic reagents

*n*-Butyllithium (2.5 M in hexanes), and *tert*-butyllitium (1.7 M in pentane) were commercial reagents. Vinyllithium was generated by the reaction of tetravinyltin with *tert*-butyllithium as previously described [6,13]. Briefly, a solution of tetravinyltin (0.7 mL, 3.2 mmol) in tetrahydrofuran (10 mL) was treated dropwise at -70 °C for 5 min with *tert*-butyllithium (7.4 mL, 12.6 mmol), and the mixture was stirred for an additional 10 min at -70 °C before use. α-Lithiostyrene was generated by dropwise addition of *n*-butyllithium (1.2 mL, 3.0 mmol) to a stirred solution of α-bromostyrene (0.43 mL, 3.0 mmol) in tetrahydrofuran (3.0 mL) at -70 °C and the mixture was kept at -70 °C for 5 min before use. Methylmagnesium bromide (3.0 M in diethyl ether), and phenylmagnesium bromide (1.0 M in tetrahydrofuran) were commercial reagents. 

*2-Chloro-4-vinylpyrimidine* (**2**). This compound was synthesized as reported by us previously [7] and characterized as follows (this characterization has not been published in detail previously): an oil, yield 50%; ^1^H-NMR (CDCl_3_): δ 8.57 (d, *J* = 5.0 Hz, 1H), 7.22 (d, *J* = 5.0 Hz, 1H), 6.70 (m, 1H), 6.52 (m, 1H), 5.80 (m, 1H); ^13^C-NMR (CDCl_3_): δ 165.5, 161.6, 159.9, 133.9, 125.3, 116.5. High-resolution MS (ESI, positive ion mode): calcd. for C_6_H_5_^35^ClN_2_ (M^+^ + 1), *m/z* 141.0218; found *m/z* 141.0220.

*2-Chloro-4-(1-phenylvinyl)pyrimidine* (**3**). A solution of α-lithiostyrene (3.0 mmol) in tetrahydrofuran (3 mL) was generated as described above. This solution was treated at once with one portion of a solution of 2-chloropyrimidine (**1**, 0.21 g, 1.8 mmol) in tetrahydrofuran (2 mL). After stirring for an additional 5 min at -70 °C the mixture was quenched with water (0.1 mL) in tetrahydrofuran (1 mL), and the stirring was continued while the temperature was allowed to rise to 0 °C. After treatment with a solution of 2,3-dichloro-5,6-dicyanoquinone (DDQ, 0.7 g, 3 mmol) in tetrahydrofuran (4 mL) the mixture was stirred at 23 °C for 5 min and then cooled to 0 °C, treated with a cold solution of sodium hydroxide (4 M, 2 mL, 8 mmol) and extracted at 0 °C with hexanes/tetrahydrofuran (1:1, 20 mL). The extract was dried over magnesium sulfate, filtered and concentrated under reduced pressure. The residue was purified by chromatography eluting with hexanes/dichloromethane (1:1) to provide **3** as colorless oil in 58% yield (0.23 g); ^1^H-NMR (CDCl_3_): δ 8.53 (d, *J* = 5.2 Hz, 1H), 7.40 (m, 3H), 7.31 (m, 2H), 7.09 (d, *J* = 5.2 Hz, 1H), 6.48 (m, 1H), 5.77 (m, 1H); ^13^C-NMR (CDCl_3_): δ 167.7, 161.5, 159.8, 145.9, 138.1, 128.6, 128.5, 128.4, 123.1, 117.5. High-resolution MS (ESI, positive ion mode): calcd. for C_12_H_10_^35^ClN_2_ (M^+^+ 1), *m/z* 217.0533; found *m/z* 217.0526.

### 3.3. General procedure for the syntheses of ***4*** (R = Ph, Nu = OMe, Scheme 1) and ***5–12***

A solution of **2** or **3** (0.4 mmol) in toluene (3.0 mL) was treated with nucleophile (amine, MeONa, EtSNa, PhSNa, 0.4 mmol). The mixture was stirred at room temperature overnight or at 90 °C for 2 h, then cooled to room temperature, basified with sodium carbonate and extracted with ethyl ether (3 × 15 mL). The extract was dried over magnesium sulfate, and concentrated *in vacuo* to provide crude 2-chloro-4-substituted pyrimidines **4** that without purification were used for the reaction with *N*-methylpiperazine. Thus, a solution of crude compound **4** and *N*-methylpiperazine (0.14 mL, 1.2 mmol) in toluene (4.0 mL) was heated to 80 °C until a TLC analysis on silica gel eluting with hexanes/ethyl ether (4:1) showed the absence of **4** (several hours). The mixture was cooled to room temperature, basified with sodium carbonate and extracted with ethyl ether (3 × 15 mL). The extract was dried over magnesium sulfate, and concentrated under reduced pressure to provide crude compounds **5–12** that were purified by chromatography eluting with hexanes/ethyl ether/methanol (16:3:1). A single compound **4** (R = Ph, Nu = OMe in Scheme 1) was purified by chromatography in a similar manner.

*2-Chloro-4-(2-methoxy-1-phenylethyl)pyrimidine* (**4**, *R = Ph, Nu = OMe*)*.* This compound was obtained as an oil in 25%; ^1^H-NMR (CDCl_3_): δ 8.47 (d, *J* = 5.2 Hz, 1H), 7.29 (m, 4H), 7.26 (m, 1H), 7.12 (d, *J* = 5.2 Hz, 1H), 4.31 (dd, *J* = 8.8, 5.6 Hz, 1H), 4.20 (t, *J* = 8.8 Hz, 1H), 3.88 (dd, *J* = 8.8, 5.6 Hz, 1H), 3.35 (s, 3H); ^13^C-NMR (CDCl_3_): δ 173.6, 161.3, 159.3, 138.4, 128.9, 128.3, 127.6, 119.5, 74.5, 59.1, 53.2. High-resolution MS (ESI, positive ion mode): calcd. for C_13_H_13_^35^ClN_2_O (M^+^ + 1), *m/z* 249.0795; found *m/z* 249.0784. 

*N,N-Dimethyl-2(2-(4-methylpiperazino)pyrimidin-4-yl)ethanamine* (**5**). This compound was obtained as a brown oil in 55% yield; ^1^H-NMR (CDCl_3_): δ 8.20 (d, *J* = 4.8 Hz, 1H), 6.41 (d, *J* = 4.8 Hz, 1H), 3.86 (t, *J* = 5.2 Hz, 4H), 2.77–2.74 (m, 2H), 2.71–2.69 (m, 2H), 2.48 (t, *J* = 5.2 Hz, 4H), 2.35 (s, 3H), 2.31 (s, 6H); ^13^C-NMR (CDCl_3_): δ 169.2, 161.7, 157.4, 109.4, 57.9, 54.9, 46.2, 45.2, 43.6, 35.7. High-resolution MS (ESI, positive ion mode): calcd. for C_13_H_23_N_5_ (M^+^ + 1), *m/z* 250.2007; found *m/z* 250.2032.

*N-(2-(2-(4-Methylpiperazino)pyrimidin-4-yl)ethyl)butan-1-amine* (**6**). This compound was obtained as an oil in 46% yield; ^1^H-NMR (CDCl_3_): δ 8.19 (d, *J* = 5.0 Hz, 1H), 6.38 (d, *J* = 5.0 Hz, 1H), 3.84 (t, *J* = 5.0 Hz, 4H), 2.97 (t, *J* = 6.6 Hz, 2H), 2.77 (t, *J* = 6.6 Hz, 2H), 2.63 (t, *J* = 7.2 Hz, 2H), 2.46 (t, *J* = 4.8 Hz, 4H), 2.34 (s, 3H), 1.49–1.43 (m, 2H), 0.93–0.89 (m, 2H), 0.91 (t, *J* = 7.2 Hz, 3H); ^13^C- NMR (CDCl_3_): δ 169.4, 161.7, 157.3, 157.1, 109.3, 54.9, 49.5, 48.1, 46.2, 43.6, 37.7, 20.5, 14.0. High-resolution MS (ESI, positive ion mode): calcd. for C_15_H_26_N_5_ (M^+^ + 1), *m/z* 278.2340; found *m/z* 278.2345.

*2-Methyl-N-(2-(2-(4-methylpiperazino)pyrimidin-4-yl)ethyl)propan-2-amine* (**7**). This compound was obtained as an oil in 38% yield; ^1^H NMR (CDCl_3_): δ 8.20 (d, *J* = 4.8 Hz, 1H), 6.40 (d, *J* = 5.0 Hz, 1H), 3.85 (t, *J* = 3.2 Hz, 4H), 2.97–2.92 (m, 2H), 2.79–2.75 (m, 2H), 2.48 (t, *J* = 5.0 Hz, 4H), 2.34 (s, 3H), 1.11 (s, 9H); ^13^C NMR (CDCl_3_): δ 169.5, 161.6, 157.4, 109.4, 55.0, 50.2, 46.2, 43.6, 40.8, 38.4, 29.0. *High-resolution* MS (ESI, positive ion mode): calcd. for C_15_H_26_ N_5_ (M^+^ + 1), *m/z* 278.2345; found *m/z* 278.23.

*N-Benzyl-2-(2-(4-methylpiperazino)pyrimidin-4-yl)ethanamine tetrahydrobromide* (**8**). This salt was obtained in 62% yield; m.p. 110–112 °C; ^1^H-NMR (DMSO-*d*_6_): δ 10.0 (bs, 1H), 8.96 (bs, 1H), 8.37 (d, *J* = 4.8 Hz, 1H), 7.58–7.55 (m, 2H), 7.49–7.42 (m, 3H), 6.73 (d, *J* = 5.4 Hz, 1H), 4.68 (d, *J* = 14.4 Hz, 2H), 4.24 (t, *J* = 5.4 Hz, 2H), 3.55–3.50 (m, 2H), 3.39–3.29 (m, 4H), 3.06 (t, *J* = 7.6 Hz, 4H), 2.86 (bs, 3H); ^13^C-NMR (CDCl_3_): δ 171.3, 163.2, 158.3, 143.2, 128.3, 128.1, 125.9, 110.2, 55.3, 54.2, 46.3, 44.0, 39.3, 34.2. MS (ESI) *m/z* 312 (M^+^ + H). *Anal.* Calcd for C_18_H_25_N_5_·4HBr·H_2_O: C, 33.10; H, 4.78; N, 10.72. Found: C, 33.43; H, 5.10; N, 10.66. 

*4-(2-Methoxyethyl)-2-4-methylpiperazino)pyrimidine trihydrobromide* (**9**). The free base was obtained as an oil in 55% yield; ^1^H-NMR for the free base (CDCl_3_): δ 8.22 (d, *J* = 5.0 Hz, 1H), 6.44 (d, *J* = 5.0 Hz, 1H), 3.87 (t, *J* = 5.0 Hz, 4H), 3.77 (t, *J* = 6.8 Hz, 2H), 3.37 (s, 3H), 2.85 (t, *J* = 6.8 Hz, 2H), 2.48 (t, *J* = 4.8 Hz, 4H), 2.36 (s, 3H); ^13^C-NMR (CDCl_3_): δ 168.1, 161.7, 157.4, 109.4, 58.7, 55.0, 46.2, 43.6, 38.0, 29.7. High-resolution MS for the free base (ESI, positive ion mode): calcd. for C_12_H_20_ N_4_O (M^+^ + 1), *m/z* 237.1705; found *m/z* 237.1715. *Anal.* calcd for: calcd for C_12_H_20_N_4_O·3HBr: C, 30.09; H, 4.24; N, 11.70. Found: C, 29.86; H, 4.34; N, 12.12.

*4-(2-(Ethylthio)ethyl)-2-(4-methylpiperazino)pyrimidine* (**10**). This compound was obtained as an oil in 25% yield; ^1^H-NMR (CDCl_3_): δ 8.22 (d, *J* = 5.0 Hz, 1H), 6.41 (d, *J* = 5.0 Hz, 1H), 3.87 (t, *J* = 4.8 Hz, 4H), 2.92–2.90 (m, 2H), 2.88–2.86 (m, 2H), 2.60 (q, *J* = 7.4 Hz, 2H), 2.49 (t, *J* = 4.8 Hz, 4H), 2.36 (s, 3H), 1.29 (t, *J* = 7.4 Hz, 3H); ^13^C-NMR (CDCl_3_): δ 168.9, 161.7, 157.4, 109.1, 55.0, 46.2, 43.6, 38.0, 29.8, 26.0, 14.7. High-resolution MS (ESI, positive ion mode): calcd. for C_13_H_22_N_4_S (M^+^ + 1), *m/z* 267.1632; found *m/z* 267.1643. 

*N-Benzyl-2-(2-(4-methylpiperazino)pyrimidin-4-yl)-2-phenylethanamine* (**11**). This compound was obtained as an oil in 57% yield; ^1^H-NMR (CDCl_3_) δ 8.13 (d, *J* = 5.1 Hz, 1H), 7.25 (m, 10H), 6.29 (d, *J* = 5.1 Hz, 1H), 4.11 (dd, *J* = 6.4, 8.4Hz, 1H), 3.82 (m, 6H), 3.49 (dd, *J* = 8.4, 11.8Hz, 1H), 3.07 (dd, *J* = 6.4, 11.8Hz, 1H), 2.45 (m, 4H), 2.34 (s, 3H); ^13^C-NMR (CDCl_3_) δ 170.8, 161.6, 157.7, 141.1, 140.3, 128.6, 128.4, 128.3, 128.1, 127.0, 126.9, 109.7, 55.0, 53.9, 53.1, 53.0, 46.3, 43.7. High resolution MS (ESI, positive ion mode): calcd. for C_24_H_29_N_5_, (M^+^+1), *m/z* 388.2501, found *m/z* 388.2491.

*4-(2-Ethylthio)-1-phenylethyl)-2-(4-methylpiperazino)pyrimidine* (**12**). This compound was obtained as an oil in 37% yield; ^1^H-NMR (CDCl_3_) δ 8.15 (d, *J* = 5.2 Hz, 1H), 7.29 (m, 5H), 6.34 (d, *J* = 5.2 Hz, 1H), 4.01 (m, 1H), 3.88 (m, 4H), 3.48 (m, 1H), 3.06 (m, 1H), 2.48 (m, 6H), 2.35 (s, 3H), 1.22 (t, *J* = 7.2 Hz, 3H); ^13^C-NMR (CDCl_3_): δ 170.7, 161.6, 157.7, 141.7, 128.5, 128.1, 127.1, 109.4, 55.0, 53.8, 46.3, 43.7, 36.1, 26.7, 14.8. High-resolution MS (ESI, positive ion mode): calcd. for C_19_H_26_N_4_S (M^+^ + 1), *m/z* 343.1956; found *m/z* 343.1947.

*N,N-bis-{[2-(4-Methylpiperazino)pyrimidin-4-yl]ethyl}ethanamine* (**14**). Ethylamine (70% in tetrahydrofuran (0.04 mL, 0.55 mmol) was added to a solution of 2-chloro-4-vinylpyrimidine (**2**, 0.14 g, 1.0 mmol) in toluene (2.0 mL). The mixture was stirred for 48 h at 23 °C and then heated to 110 °C for 2 h after which time a TLC analysis showed the absence of **2**. The mixture was cooled to room temperature, extracted with ethyl ether (2 × 15 mL), dried over magnesium sulfate and concentrated on a rotary evaporator. The residue was purified by chromatography eluting with hexanes/ethyl ether/methanol (8:1:1) to provide **14** as a yellow oil in 38% yield (88 mg); ^1^H-NMR (CDCl_3_): δ 8.12 (d, *J* = 4.8 Hz, 2H), 6.37 (d, *J* = 4.8 Hz, 2H), 3.87 (t, *J* = 4.6 Hz, 8H), 2.91 (q, *J* = 8.0 Hz, 4H), 2.73 (q, *J* = 8.0 Hz, 4H), 2.64 (q, *J* = 7.2 Hz, 2H), 2.47 (t, *J* = 4.6 Hz, 8H), 2.35 (s, 6H), 1.04 (t, *J* = 7.2 Hz, 3H); ^13^C -NMR (CDCl_3_): δ 169.7, 162.3, 157.2, 109.4, 55.0, 51.9, 47.4, 46.3, 43.6, 35.3, 12.1. High-resolution MS (ESI, positive ion mode): calcd. for C_24_H_39_N_9_ (M^+^ + 1), *m/z* 454.3405; found *m/z* 454.3407. 

### 3.4. General procedure for pyrimidines ***15–17***

Methylmagnesium bromide (3 M in ether, 0.34 mL, 1 mmol) or phenylmagnesium bromide (1 M in tetrahydrofuran, 1.0 mL, 1.0 mmol) was added to cuprous iodide (94 mg, 0.5 mmol) in tetrahydrofuran (2 mL) at -50 °C. The mixture was stirred for 10 min at -50 °C and then treated dropwise with a solution of compound **2** (100 mg, 0.7 mmol or **3** (160 mg, 0.7 mmol) in tetrahydrofuran (3 mL). The mixture was stirred at -40 °C for 2 h, then quenched with aqueous solution of ammonium chloride (2 M, 2.0 mL) and extracted with ethyl acetate (3 × 15 mL). The extract was dried over magnesium sulfate and concentrated on a rotary evaporator. A solution of the residue and *N*-methylpiperazine (0.12 mL, 1.0 mmol) in toluene (3.0 mL) was heated to 120 °C until a TLC analysis showed the absence of the Grignard adduct (several hours). The mixture was cooled to room temperature, basified with sodium carbonate, and extracted with ethyl ether (3 × 10 mL). The extract was dried over magnesium sulfate and concentrated on a rotary evaporator. The residue was subjected to chromatography eluting with hexanes/ether/methanol (16:3:1) to provide analytically pure products **15**–**17**. 

*2-(4-Methylpiperazino)-4-propylpyrimidine* (**15**). This compound was obtained as an oil in 56% yield; ^1^H-NMR (CDCl_3_): δ 8.22 (d, *J* = 5.2 Hz, 1H), 6.46 (d, *J* = 5.2 Hz, 1H), 4.67 (d, *J* = 14.0 Hz, 2H), 3.96 (t, *J* = 14.0 Hz, 2H), 3.36–3.25 (m, 7H), 2.56 (t, *J* = 7.6 Hz, 2H), 1.75–1.70 (m, 2H), 0.97 (t, *J* = 7.6 Hz, 3H); ^13^C-NMR (CDCl_3_): δ 170.2, 160.3, 157.3, 110.0, 65.8, 60.5, 39.8, 38.6, 21.6, 13.8. High-resolution MS (ESI, positive ion mode): calcd. for C_12_H_20_ N_4_ (M^+^ + 1), *m/z* 220.1700; found *m/z* 220.1706.

*2-(4-Methylpiperazino)-4-(2-phenylethyl)pyrimidine* (**16**). This compound was obtained as an oil in 77% yield; ^1^H-NMR (CDCl_3_): δ 8.19 (d, *J* = 5.0 Hz, 1H), 7.31 (t, *J* = 7.2 Hz, 2H), 7.23–7.21 (m, 3H), 6.35 (d, *J* = 5.0 Hz, 1H), 3.88 (t, *J* = 4.8 Hz, 4H), 3.04 (q, *J* = 7.2 Hz, 2H), 2.90 (q, *J* = 7.2 Hz, 2H), 2.49 (t, *J* = 4.8 Hz, 4H), 2.37 (s, 3H); ^13^C-NMR (CDCl_3_): δ 170.0, 161.7, 157.3, 141.4, 128.4, 128.3, 125.9, 109.0, 55.0, 46.3, 43.6, 39.4, 34.2. High-resolution MS (ESI, positive ion mode): calcd. for C_17_H_22_N_4_ (M^+^ + 1), *m/z* 283.1931; found *m/z* 283.1923. 

*2-(4-Methylpiperazino)-4-(1-phenylpropyl)pyrimidine* (**17**). This compound was obtained as an oil in 40% yield; ^1^H-NMR (CDCl_3_) δ 8.13 (d, *J* = 5.2 Hz, 1H), 7.30 (m, 4H), 7.20 (m, 1H), 6.32 (d, *J* = 5.2 Hz, 1H), 3.86 (t, *J* = 5.2 Hz, 4H), 3.66 (t, *J* = 7.6 Hz, 1H), 2.46 (t, *J* = 5.2 Hz, 4H), 2.34 (s, 3H), 2.23 (m, 1H), 1.99 (m, 1H), 0.88 (t, *J* = 7.6 Hz, 3H); ^13^C-NMR (CDCl_3_) δ 172.5, 161.7, 157.4, 142.8, 128.3, 128.2, 126.5, 109.0, 55.4, 55.0, 46.3, 43.7, 27.5, 12.5. High-resolution MS (ESI, positive ion mode): calcd. for C_18_H_24_N_4_ (M + 1)^+^, *m/z* 297.2079; found *m/z* 297.2089.

### 3.5. General procedure for quinazolines ***20–26***

Synthesis of 2-chloro-4-vinylquinazoline (**19**) has been reported by us recently [7]. To a solution of **19** (190 mg, 1.0 mmol) in toluene (3.0 mL) was added the corresponding nucleophile (amine, MeONa, EtSNa, PhSNa, 1.0 mmol). The mixture was stirred at room temperature overnight or at 90 °C for 2 h, then treated at room temperature with an aqueous solution of ammonium chloride (2M, 2 mL), and extracted with ether (3 × 15 mL). The extract was dried over magnesium sulfate and concentrated under reduced pressure to provide crude 4-substituted 2-chloroquinazoline that without purification was used for the reaction with *N*-methylpiperazine. Thus, a solution of the residue after concentration and *N*-methylpiperazine (0.30 mL, 2.7 mmol) in toluene (3.0 mL) was heated to 80 °C until a TLC analysis on silica gel eluting with hexanes/ether/methanol (16:3:1) showed the absence of the intermediate product (several hours). The solution was cooled to room temperature, basified with sodium carbonate, and extracted with ethyl ether (3 × 15 mL). The extract was dried over magnesium sulfate and concentrated on a rotary evaporator to provide crude compounds **20–26**. These products were purified by chromatography eluting with hexanes/ether/methanol (5:4:1).

*N,N-Dimethyl-2-(2-(4-methylpiperazino)quinazolin-4-yl)ethanamine hydrobromide* (**20**). The free base was obtained as a yellow oil in 27% yield; ^1^H-NMR for the free base (CDCl_3_): δ 7.86 (d, *J* = 8.0 Hz, 1H), 7.60–7.57 (m, 2H), 7.18 (t, *J* = 7.2 Hz, 1H), 3.99 (t, *J* = 5.0 Hz, 4H), 3.29 (t, *J* = 7.2 Hz, 2H), 2.85 (t, *J* = 8.0 Hz, 2H), 2.51 (t, *J* = 5.0 Hz, 4H), 2.36 (s, 6H), 2.35 (s, 3H); ^13^C-NMR for the free base (CDCl_3_): δ 170.1, 158.4, 152.3, 133.3, 126.4, 124.6, 122.1, 118.6, 57.6, 55.1, 46.3, 45.5, 43.8, 32.4. High-resolution MS (ESI, positive ion mode): calcd. for C_17_H_25_N_5_ (M^+^ + 1), *m/z* 300.2175; found *m/z* 300.2188. A hydrobromide salt: m.p. 100–106 °C (dec.). *Anal.* Calcd. for C_17_H_25_N_5_·3.5HBr·H_2_O: C, 34.00; H, 5.12; N, 11.66. Found: C, 33.74; H, 5.40; N, 11.26.

*N-{2-[2-(4-Methylpiperazino)quinazolin-4-yl]ethyl}butanamine trihydrobromide* (**21**). The free base was obtained as a brown oil in 31% yield; ^1^H-NMR for the free base (CDCl_3_): δ 7.83 (d, *J* = 8.0 Hz, 1H), 7.62–7.53 (m, 2H), 7.14 (d, *J* = 8.0 Hz, 1H), 3.96 (s, 4H), 3.49 (t, *J* = 6.6 Hz, 2H), 3.30 (t, *J* = 6.6 Hz, 2H), 2.81 (t, *J* = 7.2 Hz, 2H), 2.51 (t, *J* = 4.8 Hz, 4H), 2.36 (s, 3H), 1.64–1.59 (m, 3H), 1.40–1.32 (m, 2H), 0.90 (t, *J* = 7.2 Hz, 3H); ^13^C-NMR for the free base (CDCl_3_): δ 169.1, 158.3, 152.5, 133.8, 126.6, 124.7, 122.6, 118.6, 55.2, 49.3, 46.8, 46.4, 44.1, 32.5, 31.2, 20.6, 14.0; High-resolution MS (ESI, positive ion mode): calcd. for C_19_H_29_N_5_ (M^+^ + 1), *m/z* 328.2503; found *m/z* 328.2501. A hydrobromide salt: m.p. 153–155 °C. *Anal.* Calcd. for C_19_H_29_N_5_·3HBr·3H_2_O: C, 36.04; H, 6.21; N, 11.06. Found: C, 35.89; H, 6.00; N, 11.28.

*N-Benzyl-2-(2-(4-methylpiperazino)quinazolin-4-yl)ethanamine trihydrobromide* (**22**). The free base was obtained as a yellow oil in 50% yield; ^1^H-NMR for the free base (CDCl_3_): δ 7.84 (d, *J* = 8.0 Hz, 1H), 7.64–7.56 (m, 2H), 7.33 (d, *J* = 4.4 Hz, 4H), 7.28–7.25 (m, 1H), 7.18 (t, *J* = 8.0 Hz, 1H), 3.96 (d, *J* = 4.6 Hz, 4H), 3.88 (s, 2H), 3.38 (t, *J* = 6.2 Hz, 2H), 3.22 (t, *J* = 6.2 Hz, 2H), 2.49 (t, *J* = 4.6 Hz, 4H), 2.36 (s, 3H), 2.26 (bs, 1H); ^13^C-NMR for the free base (CDCl_3_): δ 170.1, 158.4, 152.4, 140.2, 133.6, 128.6, 128.3, 127.2, 126.5, 124.7, 122.4, 118.8, 55.2, 54.2, 46.7, 46.4, 44.0, 33.7. High-resolution MS (ESI, positive ion mode): calcd. for C_22_H_27_N_5_ (M^+^ + 1), *m/z* 362.2354; found *m/z* 362.2345. A hydrobromide salt: m.p. 160–162 °C. *Anal.* Calcd. for C_22_H_27_N_5_·3HBr: C, 43.73; H, 5.00; N, 11.59. Found: C, 43.88; H, 5.48; N, 11.39.

*2-(4-Methylpiperazino)-4-(2-(4-phenylpiperazino)ethyl)quinazoline tetrahydrobromide* (**23**). The free base was obtained as a brown oil in 23% yield; ^1^H-NMR for the free base (CDCl_3_): δ 7.90 (d, *J* = 8.0 Hz, 1H), 7.66–7.58 (m, 2H), 7.31–7.27 (m, 2H), 7.21 (t, *J* = 8.0 Hz, 1H), 6.97 (d, *J* = 8.0 Hz, 2H), 6.88 (t, *J* = 7.2 Hz, 1H), 4.03 (s, 4H), 3.39 (t, *J* = 7.8 Hz, 2H), 3.27 (t, *J* = 4.8 Hz, 4H), 3.02 (t, *J* = 7.8 Hz, 2H), 2.79 (t, *J* = 4.8 Hz, 4H), 2.55 (t, *J* = 6.0 Hz, 4H), 2.39 (s, 3H); ^13^C-NMR for the free base (CDCl_3_): δ 170.2, 159.3, 152.5, 151.4, 133.6, 129.3, 126.7, 124.8, 122.5, 119.9, 118.8, 116.3, 56.6, 55.2, 53.4, 49.3, 46.3, 43.9, 31.9. High-resolution MS (ESI, positive ion mode): calcd. for C_25_H_32_N_6_ (M^+^ + 1), *m/z* 417.2751; found *m/z* 417.2767. A hydrobromide salt: m.p. 190–194 °C (dec.). *Anal.* Calcd. for C_25_H_32_N_6_•4HBr•2H_2_O: C, 38.68; H, 5.19; N, 10.83. Found: C, 38.78; H, 5.51; N, 10.46.

*4-(2-Methoxyethyl)-2-(4-methylpiperazino)quinazoline* (**24**). This compound was obtained as a yellow solid in 27% yield; m.p. 35–37 °C; ^1^H-NMR (CDCl_3_): δ 7.86 (d, *J* = 8.0 Hz, 1H), 7.61–7.55 (m, 2H), 7.18 (t, *J* = 8.0 Hz, 1H), 3.99 (t, *J* = 4.8 Hz, 4H), 3.94 (t, *J* = 7.2 Hz, 2H), 3.41–3.38 (m, 5H), 2.51 (t, *J* = 4.8 Hz, 4H), 2.35 (s, 3H); ^13^C-NMR (CDCl_3_): δ 168.9, 158.4, 152.3, 133.4, 126.4, 124.7, 122.2, 118.8, 70.5, 58.8, 55.1, 46.3, 43.8, 34.1. High-resolution MS (ESI, positive ion mode): calcd. for C_16_H_22_N_4_O (M^+^ + 1), *m/z* 287.1874; Found *m/z* 287.1872. *Anal.* Calcd. for C_16_H_22_N_4_O: C, 67.11; H, 7.74; N, 19.56. Found: C, 67.33; H, 8.05; N, 19.22.

*4-(2-(Ethylthio)ethyl)-2-(4-methylpiperazino)quinazoline* (**25**). This compound was obtained as a yellow solid in 35% yield; m.p. 38–40 °C; ^1^H-NMR (CDCl_3_): δ 7.83 (d, *J* = 8.0 Hz, 1H), 7.63–7.55 (m, 2H), 7.18 (t, *J* = 7.0 Hz, 1H), 4.00 (t, *J* = 4.8 Hz, 4H), 3.41 (t, *J* = 7.0 Hz, 2H), 3.07 (t, *J* = 8.0 Hz, 2H), 2.63 (q, *J* = 7.0 Hz, 2H), 2.51 (t, *J* = 4.8 Hz, 4H), 2.36 (s, 3H), 1.30 (t, *J* = 8.0 Hz, 3H); ^13^C-NMR (CDCl_3_): δ 169.8, 158.6, 152.5, 133.6, 126.6, 124.6, 122.4, 118.6, 55.3, 46.5, 44.0, 34.6, 29.3, 26.4, 15.0. High-resolution MS (ESI, positive ion mode): calcd. for C_17_H_24_N_4_S (M^+^ + 1), *m/z* 317.1789; Found *m/z* 317.1800; *Anal.* Calcd. for C_17_H_24_N_4_S: C, 64.52; H, 7.64; N, 17.70. Found: C, 64.71; H, 7.81; N, 17.30.

*2-(4-Methylpiperazino)-4-(2-(phenylthio)ethyl)quinazoline dihydrobromide* (**26**). This compound was obtained as a yellow oil in 50% yield; ^1^H-NMR for the free base (CDCl_3_): δ 7.71 (d, *J* = 8.4 Hz, 1H), 7.62–7.54 (m, 2H), 7.40 (d, *J* = 7.6 Hz, 2H), 7.30 (t, *J* = 7.6 Hz, 2H), 7.21 (d, *J* = 7.2 Hz, 1H), 7.14 (t, *J* = 6.8 Hz, 1H), 4.00 (s, 4H), 3.51–3.43 (m, 4H), 2.52 (t, *J* = 5.2 Hz, 4H), 2.37 (s, 3H); ^13^C-NMR for the free base (CDCl_3_): δ 169.4, 158.5, 152.5, 136.4, 133.7, 129.6, 129.2, 126.6, 126.4, 124.5, 122.4, 118.6, 55.3, 46.4, 44.0, 33.9, 31.2. High-resolution MS (ESI, positive ion mode): calcd. for C_21_H_24_N_4_S (M^+^ + 1), *m/z* 365.1791; Found *m/z* 365.1800. A hydrobromide salt: m.p. 135–137 °C. *Anal.* Calcd. for C_21_H_24_N_4_S·2HBr·H_2_O: C, 46.34; H, 5.18; N, 10.28. Found: C, 46.14; H, 5.18; N, 9.88.

## 4. Conclusions 

A novel synthetic modification of the pyrimidine and quinazoline systems involves a selective conjugate addition reaction of various *N*-, *O*-, *S*-, and *C*-centered nucleophiles across the vinyl function of 2-chloro-4-vinylpyrimidine, 2-chloro-4-(1-phenylvinyl)pyrimidine, and 2-chloro-4-vinylquinazoline followed by nucleophilic displacement of chloride in the resultant intermediate products. 

## Figures and Tables

**Scheme 3:**
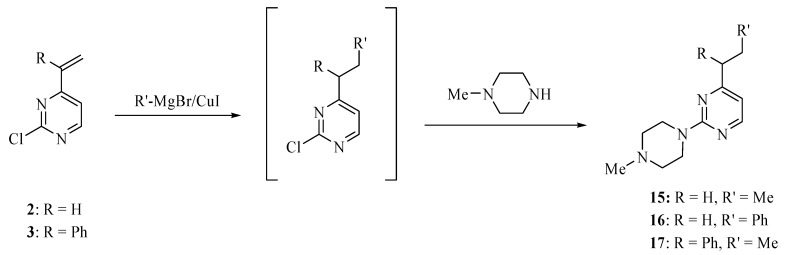
Synthesis of pyrimidines **15–17**.
